# A new passive immune strategy based on IgY antibodies as a key element to control neonatal calf diarrhea in dairy farms

**DOI:** 10.1186/s12917-020-02476-3

**Published:** 2020-07-29

**Authors:** Celina Guadalupe Vega, Marina Bok, Maren Ebinger, Lucía Alejandra Rocha, Alejandra Antonella Rivolta, Valeria González Thomas, Pilar Muntadas, Ricardo D’Aloia, Verónica Pinto, Viviana Parreño, Andrés Wigdorovitz

**Affiliations:** 1 Incuinta, Instituto Nacional de Tecnología Agropecuaria (INTA), Buenos Aires, Argentina; 2 Instituto de Virología e Innovaciones Tecnológicas, Consejo Nacional de Investigaciones Científicas y Técnicas (IVIT-CONICET), Buenos Aires, Argentina; 3 Bioinnovo S.A, Buenos Aires, Argentina; 4 El Mangrullo Farm, Buenos Aires, Argentina; 5 Servicio Nacional de Sanidad y Calidad Agroalimentaria (SENASA), Buenos Aires, Argentina; 6 Vetanco S.A, Buenos Aires, Argentina

**Keywords:** Infectious neonatal calf diarrhea, IgY antibody, Rotavirus, Passive treatment

## Abstract

**Background:**

Neonatal diarrhea remains one of the main causes of morbi-mortality in dairy calves under artificial rearing. It is often caused by infectious agents of viral, bacterial, or parasitic origin. Cows vaccination and colostrum intake by calves during the first 6 h of life are critical strategies to prevent severe diarrhea but these are still insufficient. Here we report the field evaluation of a product based on IgY antibodies against group A rotavirus (RVA), coronavirus (CoV), enterotoxigenic *Escherichia coli,* and *Salmonella* sp. This product, named IgY DNT, has been designed as a complementary passive immunization strategy to prevent neonatal calf diarrhea. The quality of the product depends on the titers of specific IgY antibodies to each antigen evaluated by ELISA. In the case of the viral antigens, ELISA antibody (Ab) titers are correlated with protection against infection in calves experimentally challenged with RVA and CoV (Bok M, et al., Passive immunity to control bovine coronavirus diarrhea in a dairy herd in Argentina, 2017), (Vega C, et al., Vet Immunol Immunopathol, 142:156–69, 2011), (Vega C, et al., Res Vet Sci, 103:1–10, 2015). To evaluate the efficiency in dairy farms, thirty newborn Holstein calves were randomly assigned to IgY DNT or control groups and treatment initiated after colostrum intake and gut closure. Calves in the IgY DNT group received 20 g of the oral passive treatment in 2 L of milk twice a day during the first 2 weeks of life. Animals were followed until 3 weeks of age and diarrhea due to natural exposure to infectious agents was recorded during all the experimental time.

**Results:**

Results demonstrate that the oral administration of IgY DNT during the first 2 weeks of life to newborn calves caused a delay in diarrhea onset and significantly reduced its severity and duration compared with untreated calves. Animals treated with IgY DNT showed a trend towards a delay in RVA infection with significantly shorter duration and virus shedding compared to control calves.

**Conclusions:**

This indicates that IgY DNT is an effective product to complement current preventive strategies against neonatal calf diarrhea in dairy farms. Furthermore, to our knowledge, this is the only biological product available for the prevention of virus-associated neonatal calf diarrhea.

## Background

Neonatal calf mortality rates are still unacceptably high on dairy farms, despite all the advances in dairy herd health and productivity [11, 25, 37]. Diarrhea is the leading cause of calf mortality [34]. Neonatal calf diarrhea is generally caused by infectious agents and is a very common disease in bovine production, leading to substantial economic losses. The most common pathogens involved are bovine group A rotavirus (RVA), bovine coronavirus (CoV*)*, *Cryptosporidium parvum, Salmonella* spp. and pathogenic *Escherichia coli*, especially in animals younger than one-month-old [3, 6, 11, 17]. Antimicrobials are often systematically used to prevent and treat neonatal calf diarrhea, even though a proper etiological diagnosis is rarely carried out. This leads to the unnecessary and excessive use of antibiotics in food animal species and the potential development of resistant bacteria and severe dysbiosis, the impairment of gut microbiome. The loss of beneficial bacteria with an overgrowth of harmful organisms will increase the risk of diseases [9]. This has prompted the ban on sub-therapeutic usage of antibiotics in many countries [14, 34]. On the other hand, diagnostic testing for the most common infectious agents associated with neonatal calf diarrhea is not always available and/or can not be performed on time. This situation highlights the need for alternative solutions to antibiotics that could also be a viable alternative strategy to control virus- and parasite-associated diarrhea, which are not sensitive to antibiotics. Several passive immune therapies based on antibodies from different sources have been proposed and tested as treatments for infectious neonatal calf diarrhea [20, 21, 23, 24, 27, 28, 41, 42]. However, there are no commercially available biological products based on passive immunity for neonatal diarrhea. IgY antibodies (Abs) are recognized as an attractive approach as they are safe, easy-to-produce, and effective for the prevention and treatment of infectious neonatal calf diarrhea. Furthermore, IgY Abs can be produced in cost-effective production systems under controlled conditions [13, 14, 36, 41, 42]. We have previously determined the IgY Ab titers to bovine RVA and CoV needed to prevent virus-associated diarrhea in newborn calves under controlled conditions resembling artificial rearing [7, 41, 42]. Based on these previous findings, we decided to develop a standardized biological product (named IgY DNT) that can be massively used in dairy farms for the prevention and treatment of infectious neonatal calf diarrhea. Several experimental field trials were performed in Argentina at dairy farms suffering a high incidence of neonatal calf diarrhea with promising results [43]. However, a controlled and daily monitored field trial to estimate the efficacy of this product was still missing.

This study aimed to determine the efficacy of IgY DNT on neonatal calf diarrhea incidence, onset, duration and etiology in dairy calves raised under regular artificial conditions during the first 3 weeks of life in a dairy herd in Argentina.

## Results

In this study, 30 newborn calves were randomly assigned to an experimental group treated with 2 L of fresh milk supplemented with 20 g of IgY DNT (10 mg/mL, *n* = 15 calves) or to a group fed only with 2 L of fresh milk during the first 2 weeks of life (*n* = 15 calves). All animals were raised following the same management protocol and care attention was provided by veterinarians and/or qualified technicians at least twice a day. The dairy farm “*El Mangrullo*” was selected because it showed a history of infectious neonatal diarrhea in the past years. During the experimental time (from birth and until the third week of life), no adverse reactions associated with the treatment were observed in any calf from IgY DNT treatment group (G1). One animal in the control group (G2) died at 10 days-of-life due to ulcerative colitis of unknown reasons. All calves remained at the dairy farm after the end of the experiment, under regular management conditions.

**Fig. 1:**
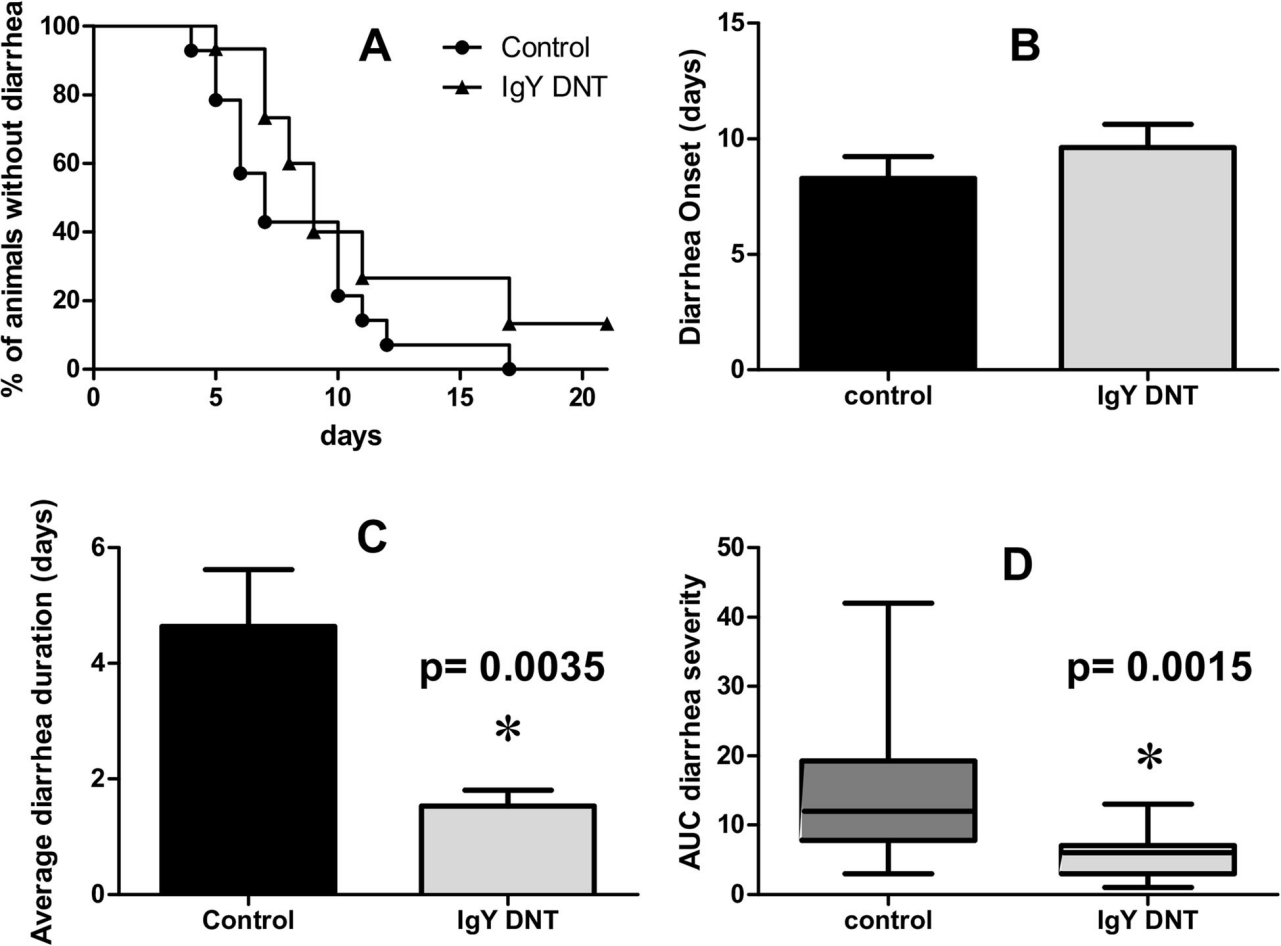
Diarrhea analysis in IgY DNT and non-treated calves. **a-** Kaplan-Meier survival curve of diarrhea for both animal groups. **b**- Diarrhea onset was considered as the first days with a fecal score equal to or higher than 2. It was recorded for each calf and the average for each experimental group was then calculated. **c**- Diarrhea duration was recorded for each calf as the number of consecutive days with a fecal score > 2. The average for each group was calculated. **d**- Diarrhea severity was estimated as the area under the fecal scores curve (AUC) for each calf was determined and then the average AUC for diarrhea was calculated for each group. The “*” indicates significant differences among the experimental groups of calves by Mann Whitney Test, with corresponding *p*-values expressed on each panel

The occurrence of neonatal diarrhea as a result of natural infection was registered daily for each calf until the third week of life (22 days). As expected, diarrhea was present in both groups of calves. However, in IgY DNT group two of the animals (2/15, 13%) did not develop diarrhea along the experimental time, while 100% (15/15) of the untreated animals showed at least one diarrhea episode (fecal score ≥ 2; Table 1), despite no significant differences were observed in the diarrhea survival curves and the mean days to diarrhea onset among groups (Fig. 1). Diarrhea severity was determined as the average of the AUC of the daily fecal score for each group of calves (Fig. 1). Animals in IgY DNT group showed significantly lower diarrhea severity (Mann Whitney test; *p* = 0.0035). This was associated with a significantly shorter duration of diarrhea, as calves in IgY DNT group presented this condition for less than 2 days in average (1.53 days, Mann Whitney test; *p* = 0.0035, Table 1), compared with control calves that presented almost three times longer diarrhea duration (4.64 days in average, Table 1). Furthermore, in IgY DNT group only one calf developed diarrhea for four consecutive days, while 38.5% (5/13) of diarrhea-affected calves showed this condition for only 1 day.

**Table 1:** Diarrhea and group A bovine rotavirus infection onset and duration

Treatment Group	% diarrhea affected calves	Diarrhea onset (days of life)	Diarrhea duration (days)	% RVA affected calves	RVA shedding onset (days of life)	RVA shedding duration (days)
G1= IgY DNT	86% (13/15)	9.26	1.53 ^a^	47% (7/15)	11.71^c^	1.00 ^e^
G2 = no Ab treatment	100% (15/15)	8.29	4.64 ^b^	47% (7/15)	7.43 ^d^	5.86 ^f^

Diarrhea was assessed daily in all calves (fecal score ≥ 2). A calf showing at least one episode of fecal score ≥ 2 was defined as diarrhea positive. Diarrhea onset and duration were computed as an average from the animals presenting this clinical condition on each experimental group. RVA was determined in all fecal samples from diarrhea-affected calves by ELISA and viral shedding onset and duration were computed as average from animals on each experimental group. Means in the same column with different superscript upper case letters differ significantly by Mann Whitney test: a-b *p*-value = 0.0035; c-d p-value = 0.0320; e-f p-value = 0.0020

**Fig. 2:**
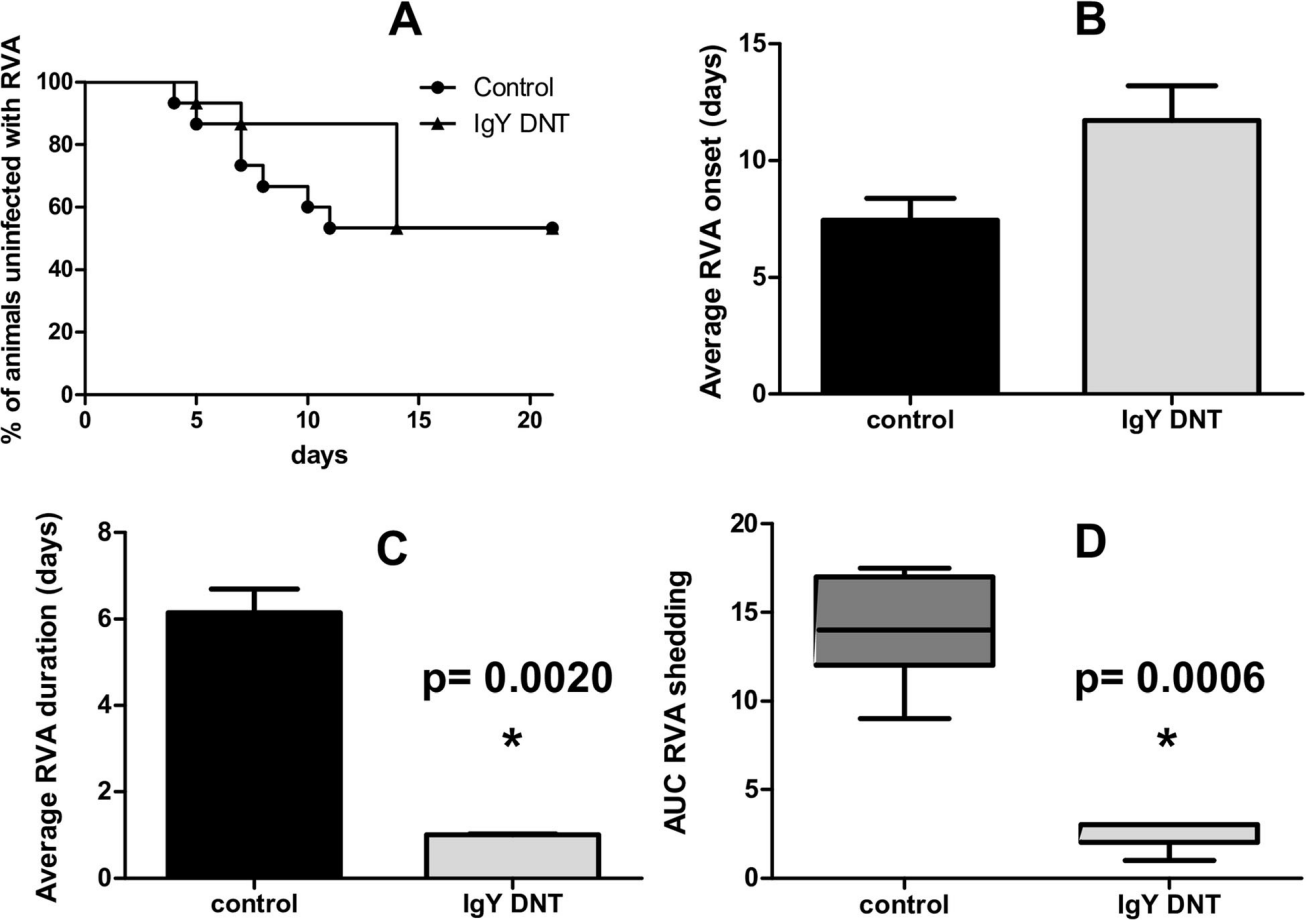
Group A Rotavirus (RVA) infection parameters in IgY DNT and control groups of calves. **a**- Kaplan-Meier survival curve of RVA infection for both animal groups. RVA was determined in all fecal samples from diarrhea affected calves by ELISA and viral shedding onset (**b**) and duration (**c**) were computed as average from animals on each experimental group. The AUC for the total of RVA shedding per group was also estimated (**d**). The “*” indicates significant differences among the experimental groups of calves by Mann Whitney Test, with corresponding p-values expressed on each panel

The presence of neonatal calf diarrhea-associated pathogens was further investigated in all fecal samples collected. No ETEC or *Salmonella* spp. were detected in any of the samples, while one calf from the control group (G2) shed CoV in feces at 24 h of life but did not develop diarrhea, so it was no further examined. Group A Rotavirus was shed by 47% (7/15) of the animals from each experimental group (Table 1). However, only 28% (2/7) of the animals in IgY group (G1) present RVA-associated diarrhea while all (100%, 7/7) calves in G2 showed this clinical condition in association with RVA detection in feces. Calves in IgY DNT group (G1) showed a trend towards a delay in the onset of RVA shedding (11.71 days) compared with animals in the control group (G2; 7.43 days). Furthermore, RVA infection survival curves did not differ significantly among groups (Fig. 2). Another important difference observed was a significantly lower viral shedding (AUC) in calves in IgY DNT treated group than in control group animals (Mann Whitney test; *p* = 0.002; Fig. 2).

A premise of the experimental design was that all the animals recruited for this study should present statistically similar Ab titers in serum to bovine RVA and CoV after colostrum intake. The geometric mean IgG1 Ab titers to RVA and CoV in calves’ sera at 1, 8, 15 and 22 days of life were similar among calves in G1 and G2 (by a mixed model for repeated measures, data not shown), even when animals in the control group (G2) developed RVA-associated diarrhea. The geometric mean IgG1 Ab titers to RVA immediately after colostrum intake (day 1 of life) were 19,700 in G1 and 28,500 in G2, while ELISA IgG1 titers to CoV were 12,400 in G1 and 13,600 in G2. These values are consistent with the expected IgG1 Ab titers in serum for calves receiving colostrum from vaccinated dams [7, 27]. As expected, ELISA IgG1 Ab titers to RVA and CoV showed a trend towards lower titers along the experimental time (22 days of life). By the end of the experiment, ELISA IgG1 Ab titers to RVA were 7800 in G1 and 10,000 in G2, while for CoV were 3100 in G1 and 3400 in G2.

## Discussion

Neonatal diarrhea is still an important concern in dairy herds that is not completely prevented with good management and sanitary practices available, likely because of the multi-factorial nature of the disease [11, 18]. In the United States, diarrhea is the most common disease condition in pre-weaned dairy calves, accounting for 56.4% of deaths [38]. There are no virus-specific therapies available, while several bacteria strains are now antibiotic-resistant due to the misuse of antibiotics in veterinary medicine [18, 34]. More than 50% of all neonatal diarrhea appears during the first week, and only 15% occur after the second week of life [4, 5]. Interestingly, half of the neonatal deaths are associated with diarrhea, while respiratory diseases seem to represent another 15% [1, 39].

Thirty newborn calves were recruited to evaluate the IgY DNT performance for controlling neonatal calf diarrhea under natural challenge. All animals in the control group (G2) developed diarrhea at least once during the first 3 weeks of life. This result demonstrates that even when high Ab titers in calves’ sera are assured, implying optimal dams’ vaccination and a proper colostrum intake, the natural challenge with infectious agents associated with neonatal diarrhea overcomes the passive immunity conferred by maternal Abs as well as the neonatal primary immune response. To our knowledge, this is the first report of a commercially available biological product based on IgY Abs to control bovine neonatal diarrhea of infectious origin. A key feature in the development of this product was the evaluation of the ELISA IgY Ab titers needed against each infectious agent and the optimization of a product containing not only these IgY Ab titers but also meeting all the microbiological requirements for oral administration to neonatal calves. The biological product developed based on IgY Abs has shown to be safe for calves while serving to reduce the incidence and severity of infectious diarrhea. This protection has shown to be dependent on virus-specific IgY Ab titers present in the product and the duration of the treatment, as it is a passive immune strategy [41, 42]. The development of IgY DNT was done considering the minimum amount of IgY Abs per dose needed to protect calves at an acceptable price for milk producers (regarding the costs of calf feeding, diarrhea treatment, calf death, dam’s reposition, etc.). This study shows that RVA-associated neonatal calf diarrhea in dairy calves can be significantly reduced with the preventive administration of a product based on virus-specific IgY Abs. Calves treated presented a delay in RVA infection, six times shorter duration of virus shedding, and seven times lower titers of virus shedding compared with the untreated control animals. This implies lower environmental contamination, reducing the chances of the virus spreading and consequent diarrhea outbreaks. Due to the experimental design that implied only the environmental challenge of animals, no conclusions can be made regarding the role of IgY DNT treatment on other infectious agents associated with neonatal calf diarrhea, as only RVA was detected and CoV was only detected once in an asymptomatic animal in this study. The antigen-detection assays performed were the ones currently used by the Diagnosis Service Unit at INTA for bacteria and viruses in diarrhea in calves. Other more sensitive assays could have been performed to determine the circulation of infectious agents in feces, like qPCR. However, the presence of significantly low amounts of viruses and/or bacteria, which are usually ubiquitous in the dairy environment, cannot be associated with diarrhea [3, 6, 8, 16–18]. Parasites were not evaluated in this study as IgY DNT does not contain IgY Abs against them. Particularly, *Cryptosporidium parvum* has been systematically detected in the last years as an infectious agent that could be associated with diarrhea in calves [4, 10, 17]. However, as it is now being systematically detected in feces of symptomatic and asymptomatic calves in Argentina [17], efforts are being made to include at least *C. parvum* in IgY DNT production.

Several passive immune therapies based on antibodies from different sources have been proposed and tested as treatments for infectious neonatal calf diarrhea but, to our knowledge, no other biological products are available in the market [19, 20, 24]. Regarding IgY Abs, there are several reports of its efficacy for neonatal calf diarrhea prevention and treatment, where IgY Abs titer shown to be critical [14]. There are some milk supplements based on IgY Abs for calves but these do not have controlled IgY Ab titers against diarrhea-associated infectious agents. It has been shown that the supplementation of the milk diet with immune colostrum significantly reduced diarrhea and delayed viral shedding onset [22, 28]. However, the development of a product based on colostrum for milk supplementation was not an industrially scalable alternative.

Another relevant finding associated with this heterologous passive treatment based on IgY Abs is that it modulates the mucosal immune response in the gut towards higher numbers of Ab secreting cells present in the duodenum and ileum of treated animals. Most of these cells are secreting IgA Abs, as has been previously reported [41, 42]. This mechanism is still unclear, as many biologically active molecules are present in eggs (as hormones and cytokines), which stimulate the local immune response [2, 26, 29, 44]. This may represent higher immune surveillance in the gut mucosa, which is one of the main places of the entrance of infectious agents causing illness during the first weeks of life in calves. Another advantage of this passive treatment is that IgY DNT is based on polyclonal IgY Abs, so it does not induce bacterial or viral resistance. As it is a passive treatment, IgY Abs are only present in the gut lumen while the treatment is maintained and do not translocate into the bloodstream. Finally, calves treated with IgY DNT presented a healthier general condition and hair coat compared with control ones after veterinary examination, suggesting a better nutritional and immunological status, as has been previously reported [41, 42].

Here we report a useful product for the prevention of neonatal calf diarrhea that can be administered as an oral passive immune strategy during the first 2 weeks of life of dairy calves reared under artificial conditions. Today, efforts should be made in the prevention of neonatal diarrhea as it is better than cure from the viewpoint of not only productivity but also animal welfare.

## Conclusions

The systematic immunization of pregnant cows with good quality vaccines, the monitoring of colostrum intake by calves and the administration of IgY DNT during the first 2 weeks of life could be key factors to prevent neonatal calf diarrhea in dairy farms, reducing the unnecessary use of antibiotics and preventing morbidity and mortality due to this clinical condition.

## Methods

### Ethics statement

The protocol for animal management met the requirements of the Institutional Committee for Experimental Animals Welfare and Management (CICUAE), from INTA (protocol number 24/2010 calves and 20/2011 for chickens). All calves were subject to veterinary assistance following regular management protocols from the dairy farm “*El Mangrullo*”.

### IgY DNT production and quality control

Five hundred Lohmann Brown Classic laying hens were immunized intramuscularly in the breasts with commercially available vaccines containing bovine RVA, CoV Mebus and *E. coli* J5 and *Salmonella Dublin* bacterin cultures (Neonatal Block, Vetanco S.A.) under veterinary assistance. No adverse effects on vaccination were observed in any of the animals. Hens were immunized following an optimized program developed especially for IgY DNT production that has been reported elsewhere [42]. Eggs were collected daily and processed in a spray-dryer machine (FlexPump, Galaxie S.A.) to obtain a powder-based product. The quality control of every batch of this product included ELISA IgY Ab titers against RVA, CoV, and the bacterins from *E. coli* J5 and *Salmonella Dublin*, and microbiological control of total aerobic microorganisms, yeasts, and fungus in compliance with the United States Pharmacopeia –USP NF- recommendations [40]. For its acceptance, each batch should independently present an ELISA IgY Ab titer to RVA ≥2048, and an ELISA IgY Ab titer ≥64 for CoV, *E. coli* J5 and *Salmonella Dublin* in a solution prepared with 40 g of IgY DNT diluted in 2 L of Ab-free milk (20 mg/mL)*.* It also must be free of *E. coli* and *Salmonella* sp. and show less than 10,000 colony forming units (CFU)/g of aerobic microorganisms and less than 100 CFU/g of yeasts and fungus. This powder can be stored at room temperature for up to 12 months [42]. All hens remained at Bioinnovo S.A. facilities after the end of the experiment.

### Experimental design

This study was carried out in a dairy farm named “*El Mangrullo*”, located in the Northeastern of Buenos Aires Province, Argentina. The farm has 612 milking cows. A total of 30 newborn Holstein female and male calves were recruited for this study between September and November 2016. Calves were born to healthy cows vaccinated with commercial vaccines for neonatal calf diarrhea and pneumonia prevention twice in the last term of gestation. Calves were artificially fed with no less than 2 L of colostrum obtained from their dams in the first 4 h of life and a minimum of 10% their weight (around 4–5 L) of this colostrum within the first 24 h of life. After the first 24 h and up to the third week of life, calves were fed with two liters of fresh bovine milk from the dairy facilities (not pasteurized), twice a day. Only calves born at term to healthy vaccinated cows, independently of their sex, were included in this study. Calves born in dystocic delivery were not recruited for this experiment. From the third week on, the daily milk intake was of six litters in total, divided in two intakes of three litters each. All calves were ear-tagged during the first day of life. The treatment assignment was performed by randomly splitting all the ear tag numbers into two groups. Animals were then assigned to the corresponding experimental group after veterinary examination: **G1- IgY DNT** = calves fed with 2 L of milk supplemented with 20 g of IgY DNT powder (*n* = 15); and **G2 - control** = calves fed with 2 L of non-supplemented milk (*n* = 15). The passive treatment started after the first 24 h of life, to assure its administration after gut closure, and was maintained for the first 2 weeks of life (14 days in total). The supplementation of 2 L of milk with 20 g of IgY DNT (10 mg /mL) (G1) resulted in a final ELISA IgY Ab titer of 4096 to bovine RVA (UK G6P[5] strain); 64 to bovine CoV (Mebus strain), 64 to enterotoxigenic *E. coli* (ETEC) and 64 to *Salmonella* spp. (*S.* Dublin). Animals were allocated in special cages with open space and a house to protect them from weather conditions, one animal per cage. Calves were examined daily for the development of diarrhea, from birth to the third week of life (22 days-of-life). To estimate diarrhea severity, fecal consistency was scored blindly by qualified technicians as follows: 0 = normal; 1 = pasty; 2 = semi-liquid; 3 = liquid; considering a score equal or greater than 2 as diarrhea [28]. If calves developed diarrhea, fecal samples were taken daily until the cessation of clinical symptoms. Otherwise, fecal and serum samples were taken and rectal temperatures recorded weekly, starting from the first day of life and then at days 8, 15, and 22. The area under the curve (AUC) of the fecal scores for each calf was determined and then the average and median AUC for diarrhea was calculated for each group. Animals were treated with rehydrating salts, antibiotics, and antipyretics if needed after veterinarian examination. All calves remained at the dairy farm after the end of the experiment, under regular management conditions. No long-term effect that could be associated with this experiment was observed.

### Detection of infectious agents associated with neonatal calf diarrhea

Virus shedding (RVA and CoV) was detected in fecal samples using antigen-capture ELISAs as previously described [12, 33]. The AUC for daily virus shedding titers for each calf was first determined and then the average and median AUC for virus shedding was calculated for the group. The presence of *E. coli* and *Salmonella sp.* was evaluated in fecal samples by selective bacteriological culture media. For *E. coli* detection, fecal samples were cultured on MacConkey agar plates and Gram staining was performed on isolated lactose-positive colonies. Gram-negative bacteria were biochemically characterized as belonging to the *Enterobacteriaceae* family, *Escherichia* genus, according to Bergey’s Manual. Colonies compatible with *E. coli* were transferred to tubes containing minimal agar medium. PCR technique was performed as described by Siqueira and colleagues [32]. The *E. coli* strains were classified in Shiga toxin pathotypes based on their specific virulence genes (Sta). One calf was positive to ETEC if isolates were found to be positive for one of the virulence genes reported elsewhere [30, 31]. For *Salmonella* enrichment, fecal samples were 1:10 diluted in tetrathionate (TT) broth and cultured at 37 °C for 48 h and later plated in xylose lysine deoxycholate agar with tergitol 4 (XLDT4 agar) for another 24 h at 37 °C. Suspicious colonies were subject to biochemical confirmation [35].

### Evaluation of bovine antibodies to viruses

Serum IgG Abs to bovine RVA (UK G6P[5] strain) and to CoV (Mebus strain) were measured by ELISA. Virus-specific IgG Abs were detected by indirect ELISA using the reagents and protocols previously described [6, 7, 27, 41].

### Statistical analysis

The sample size was estimated considering historical neonatal diarrhea incidence in El Mangrullo farm and the maximum number of calves to be born during the experimental time proposed. For all the parameters analyzed, the statistical power was equal or above 0.8 (with a significance level of 5%), except for diarrhea onset. Kaplan Meier survival curves of the percentage of calves with diarrhea and the percentage of animals infected with RVA during the experimental time were compared by Log-rank Mantel-Cox Test (non-parametric) using GraphPad Prism version 5.01 for Windows (GraphPad Software, www.graphpad.com). Fisher’s exact test was used to compare proportions of calves with diarrhea and virus shedding between groups. For variables like days to onset, duration, and severity of diarrhea and virus shedding, where the assumptions of normality and/or homoscedasticity were not met, the Mann Whitney rank sum U-test (non-parametric) was used for comparisons between groups. IgG1 Ab titers to RVA and CoV were log10-transformed before statistical analysis. The curves of the kinetics of Ab response to RVA and CoV were analyzed using a mixed model for repeated measures and Bonferroni method for multiple comparisons, considering “group treatment” and “time” as fix effects and “calf” as a random variable (varIdent) to take into account intrinsic differences among calves and including an auto-correlation structure of variance-covariance matrix (corAr_1_). All these statistical analyses were conducted using InfoStat [15]. In all cases, the significance level was established at 5%.

## Data Availability

The datasets used and/or analyzed during the current study are available from the corresponding author on reasonable request.

## Abbreviations

RVA: Group A rotavirus
CoV: Coronavirus
Ab: Antibody
ELISA: Enzyme-linked immunosorbent assay
AUC: Area under the curve
CFU: Colony-forming units
ETEC: Enterotoxigenic *Escherichia coli*
TT: Tetrathionate broth
XLDT4: Xylose lysine deoxycholate agar with tergitol 4
PCR: Polymerase chain reaction
INTA: National Institute of Agricultural Research / Instituto Nacional de Tecnología Agropecuaria
IVIT: Virology and Technological Innovation Institute / Instituto de virología e Innovación Tecnológica
CONICET: National Scientific and Technical Research Council / Consejo Nacional de Investigaciones Científicas y Técnicas
SENASA: National Food Safety and Quality Service / Servicio Nacional de Sanidad Agropecuaria
DVM: Doctor in veterinary medicine
